# Identification and Quantification of Proliferating Cells in Skeletal Muscle of Glutamine Supplemented Low- and Normal-Birth-Weight Piglets

**DOI:** 10.3390/cells12040580

**Published:** 2023-02-11

**Authors:** Elke Albrecht, Yaolu Zhao, Miriama Sciascia, Cornelia C. Metges, Steffen Maak

**Affiliations:** 1 Research Institute for Farm Animal Biology (FBN), Institute of Muscle Biology and Growth, 18196 Dummerstorf, Germany; zhaoyl@scau.edu.cn (Y.Z.); maak@fbn-dummerstorf.de (S.M.); 2 National Engineering Research Center for Breeding Swine Industry, College of Animal Science, South China Agricultural University, Guangzhou 510642, China; 3 Research Institute for Farm Animal Biology (FBN), Institute of Nutritional Physiology “Oskar Kellner”, 18196 Dummerstorf, Germany; sciascia@fbn-dummerstorf.de (M.S.); metges@fbn-dummerstorf.de (C.C.M.)

**Keywords:** muscle satellite cell, BrdU, PAX7, preadipocyte, DLK1, immunohistochemistry

## Abstract

One way to improve the growth of low-birth-weight (LBW) piglets can be stimulation of the cellular development of muscle by optimized amino acid supply. In the current study, it was investigated how glutamine (Gln) supplementation affects muscle tissue of LBW and normal-birth-weight (NBW) piglets. Longissimus and semitendinosus muscles of 96 male piglets, which were supplemented with 1 g Gln/kg body weight or alanine, were collected at slaughter on day 5 or 26 post natum (dpn), one hour after injection with Bromodeoxyuridine (BrdU, 12 mg/kg). Immunohistochemistry was applied to detect proliferating, BrdU-positive cells in muscle cross-sections. Serial stainings with cell type specific antibodies enabled detection and subsequent quantification of proliferating satellite cells and identification of further proliferating cell types, e.g., preadipocytes and immune cells. The results indicated that satellite cells and macrophages comprise the largest fractions of proliferating cells in skeletal muscle of piglets early after birth. The Gln supplementation somewhat stimulated satellite cells. We observed differences between the two muscles, but no influence of the piglets’ birth weight was observed. Thus, Gln supplements may not be considered as effective treatment in piglets with low birth weight for improvement of muscle growth.

## 1. Introduction

Adapted feeding strategies, in particular with amino acid supplementation, have been applied to improve the growth and muscle development of low-birth-weight (LBW) piglets [1,2,3,4]. Improved growth requires increased protein accretion, enabled by higher protein synthesis rates and reduced protein degradation [5]. Studies have shown that protein synthesis can be stimulated by amino acid supplementation in all tissues, including skeletal muscle [6], but the effects decrease with age [7]. Optimal protein supply during the first days of life was hypothesized to help LBW piglets by compensating the delayed growth by stimulating proliferation of muscle satellite cells, differentiation to myoblasts and fusion with muscle fibers to improve muscle fiber growth [8]. Indeed, our previous study indicated a stimulation effect of glutamine on undefined cell types within muscle tissue of 5 d-old piglets [8]. Glutamine is a precursor for the synthesis of purine and pyrimidine nucleotides and promotes cell proliferation, but it also provides energy, when metabolized to glutamate [9,10]. As a precursor for arginine synthesis, it is important for the optimal growth of neonatal piglets [11].

Different cell types within the muscle tissue may have benefited from the glutamine supplementation, but it was more or less a speculation that most of the proliferating cells were satellite cells. Therefore, a cell culture model was applied to test the effect of glutamine on porcine satellite cells, which indicated that glutamine stimulated the proliferation of satellite cells from LBW piglets more than from normal-birth-weight (NBW) piglets [8]. To verify the importance of this effect in muscle tissue, it would be necessary to identify and quantify proliferating satellite cells. The best-established marker for satellite cells is Paired box transcription factor 7 (PAX7), which is always expressed in quiescent satellite cells [12]. This transcription factor regulates muscle growth in the early phase of cell proliferation [13] and restricts the differentiation of muscle stem cells [14]. Activation of satellite cells and commitment to the myogenic lineage is regulated and indicated by expression of MYOD1 (Myoblast determination protein 1) [15]. Myogenin is a transcription factor involved in the terminal differentiation of myoblasts to myotubes [16].

Preadipocytes, another important cell type for muscle development, cannot easily be detected. Protein delta homolog 1 (DLK1), which is also known as preadipocyte factor 1 (Pref-1), is well known for its inhibitory effect on differentiation of preadipocytes into mature adipocytes [17]. In cattle, DLK1 seems to be a candidate for specific detection of preadipocytes [18]. It was shown to be down-regulated during adipogenic differentiation in cell culture models [19,20]. However, it is widely expressed during embryogenesis [21] and was also detected in developing myofibers and associated satellite cells [22]. In mice, a dual role of DLK1 was described, playing a vital role in myogenesis and in adipogenesis [23]. Recently, Fu et al. [24] reported a similar role in porcine myogenesis and skeletal muscle development. Therefore, DLK1 may be considered as a stem cell marker in porcine muscle tissue in accordance with Zhang et al. [25]. In the early stage of adipocyte differentiation, when cells start lipid deposition and lose their ability to proliferate, DLK1 is no longer detectable; instead, the transcription factor CEBPB is abundant in the nucleus [26].

We hypothesized that a large portion of the proliferating cells in the muscle tissue of piglets were satellite cells, in particular, during the early postnatal phase when muscle grows fast, and that satellite cells were activated or stimulated by Gln supplementation. Therefore, the current study aimed to quantify proliferating satellite cells in muscle cross-sections of 5- and 26-day-old piglets with low and normal birth weight under glutamine supplementation and to identify some of the other proliferating cell types.

## 2. Materials and Methods

### 2.1. Animals and Sampling

All experimental procedures and animal care were carried out in agreement with the European Convention for the Protection of Vertebrate Animals used for Experimental and Other Scientific Purposes (2010/63/EU) and were approved by the responsible State Office for Agriculture, Food Safety and Fisheries Mecklenburg-Western Pomerania, Germany (permission No. 7221.3-1-026/16).

Ninety six male German Landrace piglets, which were generated in the experimental pig facility of the Research Institute for Farm Animal Biology (FBN), Dummerstorf, Germany, were used in this study: 48 pairs of siblings with either low (LBW, 0.8–1.2 kg; below the lowest birth weight quartile of the FBN experimental pig herd) or normal birth weight (NBW, 1.4–1.8 kg; birth weight control). Details of the experiment were reported earlier [4,27,28]. In this study, piglets were supplemented with glutamine (Gln, 1 g/kg body weight) or an isonitrogeneous amount of alanine (Ala, 1.22 g/kg body weight) from day 1 to day 12 after birth. Thus, four groups were generated: LBW-ALA, LBW-GLN, NBW-ALA, and NBW-GLN. Twelve piglets per group were slaughtered at day 5 or 26 post natum (dpn), and tissue of *Musculus longissimus dorsi* (MLD) and *Musculus semitendinosus* (MST) was collected from the left side of the carcass. Piglets were injected intraperitoneally with Bromodeoxyuridine (BrdU, 12 mg/kg body weight; Roche, Mannheim, Germany) one hour before slaughter to enable in situ detection of cell proliferation in different tissues.

### 2.2. Immunohistochemistry

Sections from MLD and MST of all 96 animals were cut 10 µm thick with a cryostat microtome (CM3050 S, Leica, Bensheim, Germany). The sections were used for double staining with antibodies against PAX7 (DSHB, Univ. Iowa, USA) and BrdU (#11170376001, Sigma-Aldrich, Munich, Germany). Sections were fixed in 4% paraformaldehyde solution in phosphate-buffered saline (PBS) for 20 min and subsequently washed 2 × 5 min with PBS and permeabilized with 0.1% TritonX-100 (Sigma-Aldrich, Munich, Germany) in PBS (PBST) for 10 min. Nonspecific bindings of the secondary antibody were blocked with 10% normal goat serum (NGS) in PBST for 15 min, at room temperature (RT). Slides were incubated with the primary antibody against PAX7 (1:100 in PBST incl. 1% NGS) over night, at 4 °C, in a humidity chamber. After 3 × 10 min washing with PBST, slides were incubated with an Alexa Fluor 594 goat anti-mouse IgG (H + L) secondary antibody (1: 500 in PBST, A-11032, Thermo Fisher Scientific, Schwerte, Germany) for 45 min, at room temperature, in the dark. The slides were then incubated with 2N HCl, at 37 °C, for 60 min to denature DNA and thereafter washed 3 × 5 min with PBST. Slides were incubated overnight, at 4 °C, with the antibody against BrdU (1:100 in PBST incl. 1% NGS) in a humidity chamber. After washing 3 × 10 min with PBST, slides were incubated with the secondary antibody (Alexa Fluor 488 goat anti-mouse IgG1, 1: 1000 in PBST, A-21121, Thermo Fisher Scientific, Schwerte, Germany) for 45 min, at RT, in the dark and then washed 1 × 5 min with PBST, 2 × 5 min with PBS and 1 × 10 min with distilled water and covered with ProLong Diamond Antifade Mountant (Thermo Fisher Scientific, Schwerte, Germany) and coverslips (Roth, Karlsruhe, Germany). The secondary antibody Alexa Fluor 488 goat anti-mouse IgG1, which was used to detect the BrdU primary antibody, was also able to detect the PAX7 antibody in a single immunostaining. However, when PAX7 was detected first with Alexa Fluor 594 goat anti-mouse IgG (H + L), there was no additional staining with Alexa Fluor 488 goat anti-mouse IgG1 (see Supplementary Figure S1). Thus, specific, independent fluorescence signals of PAX7 and BrdU were generated for analysis (see Supplementary Figure S2).

For double staining with DLK1 or CD163 and BrdU, the sections were first incubated with DLK1 (1:100, #21682 Abcam, Cambridge, UK) or CD163 (1:100, MCA1853, AbD Serotec, BioRad, Munich, Germany) for 2 h, detected with Alexa Fluor 594 goat anti-rabbit IgG or Alexa Fluor 594 goat anti-mouse IgG (H + L) (1: 1000 in PBST, Thermo Fisher Scientific, Schwerte, Germany), respectively, then a shorter denaturation step with 2N HCl, at 37 °C, for 20 min was applied before overnight incubation with the BrdU-antibody (1:100). After secondary antibody incubation and washing, slides were covered with Roti Mount Fluor Care + Dapi (HP20.1, Roth, Karlsruhe, Germany) to enable detection of all nuclei. To ensure specific, independent staining of CD163 and BrdU, the binding of both secondary anti-mouse IgG antibodies to the CD163 primary antibody was tested. The Alexa Fluor 488 goat anti-mouse IgG1 was also able to detect it in a single immunostaining. Again, when CD163 was detected first with Alexa Fluor 594 goat anti-mouse IgG (H + L), there was no or very weak additional staining with Alexa Fluor 488 goat anti-mouse IgG1 (see Supplementary Figure S3), which did not disturb the quantitative analysis.

Double staining of PAX7 or myogenin with DLK1 was performed similarly. First, slides were incubated overnight with antibodies against PAX7 (1:100, #199010 Abcam, Cambridge, UK) or myogenin (1:100, #1835 Abcam, Cambridge, UK). After secondary antibody incubation with Alexa Fluor 594 goat anti-mouse IgG (1: 500 in PBST, Thermo Fisher Scientific, Schwerte, Germany), the antibody against DLK1 was incubated for 2 h and detected with Alexa Fluor 488 goat anti-rabbit IgG (1: 1000 in PBST, Thermo Fisher Scientific, Schwerte, Germany). Nuclei were counterstained with Hoechst 33258 (Sigma-Aldrich, Munich, Germany). Negative controls for detection of unspecific bindings of all secondary antibodies were performed by replacing the primary antibody with 10% NGS in PBST. No unspecific bindings of the secondary antibodies were observed (see Supplementary Figure S4).

### 2.3. Microscopy and Image Analysis

Immunofluorescence was detected using a Nikon Microphot SA fluorescence microscope (Nikon, Duesseldorf, Germany) and either a CC-12 (OSIS, Münster, Germany) or DP74 color camera (Evident, Hamburg, Germany). CellSens software (Evident, Hamburg, Germany) was used to count the number of PAX7+ nuclei (stained red), the number of BrdU+ nuclei (stained green) and to analyze co-localization of both. Five to six (5 dpn) or ten (26 dpn) randomly selected pictures, of approximately 1.3 and 2.6 mm², respectively, were analyzed in total for each piglet. The percentages of BrdU+ and PAX7+ nuclei of total nuclei were calculated using the number/mm² from the current analysis divided by the number of total nuclei/mm² from the former analysis of hematoxylin/eosin stained sections [4]. The percentage of proliferating satellite cells was determined as ratio between BrdU+ nuclei and PAX7+ nuclei and the percentage of satellite cells of all proliferating cells was determined reciprocally.

### 2.4. Statistical Analysis

Data were analyzed with the MIXED procedure of SAS statistical software (Version 9.4, SAS Inst., Cary, NC, USA). Fixed factors, which were included in the model, were birth weight (LBW, NBW), supplementation (Gln, Ala), age (5, 26 dpn), muscle (MLD, MST) and their respective interactions. Furthermore, sow was included as random factor. The SLICE statement was used to enable the partitioned analysis of the least-squares means (LSmeans) for the interaction between birth weight and supplementation within the same age. Tukey–Kramer test was applied to analyze pairwise differences. Values are presented as LSmeans and standard errors (SE). Differences were considered significant if *p* ≤ 0.05.

## 3. Results

Immunohistochemistry was applied to detect BrdU and PAX7 simultaneously in muscle samples of *Musculus longissimus dorsi* (MLD) and *Musculus semitendinosus* (MST) of all piglets at 5 and 26 dpn, and the number of stained cells and co-localization was quantified with image analysis. Both BrdU (green) and PAX7 (red) were localized in nuclei as expected (Supplementary Figure S2) and showed individual staining patterns. The overlay of both (Supplementary Figure S2) confirmed nuclei had either one (green or red) or both labels (yellow), indicating specificity and no cross-reactivity of the antibodies, which was a prerequisite for the subsequent analysis.

**Figure 1:**
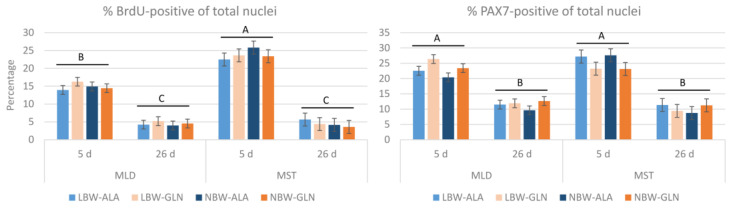
Content of BrdU- and PAX7-positive nuclei among all nuclei in muscle cross-sections of *Musculus longissimus dorsi* (MLD) and *Musculus semitendinosus* (MST) of piglets at 5 and 26 dpn (*n* = 12 per group). Total nuclei numbers were obtained from hematoxylin/eosin stained muscle sections of a former study [4]. Values represent LSmeans with standard errors in error bars. ^A–C^ indicate significant differences among ages and muscles (*p* ≤ 0.05, Tukey–Kramer test).

First, the percentage of BrdU+ of total nuclei was determined (Figure 1). The values were higher for MST than MLD at 5 dpn (23.8 ± 0.9% vs. 14.9 ± 0.7%, *p* < 0.001) over groups and were lower in both muscles at 26 dpn (4.5 ± 0.7%, *p* < 0.001). No influence of birth weight or supplementation was observed (*p* = 0.89 and 0.97, respectively).

Next, it was investigated whether the proliferating cells in muscle tissue, detected by BrdU incorporation, were satellite cells. The antibody against PAX7 was used to identify muscle satellite cells (PAX7+) with immunohistochemistry. The percentage of PAX7+ of total nuclei (Figure 1) decreased (*p* < 0.001) from 23.2 ± 0.8% in MLD at 5 dpn and 25.3 ± 1.1% in MST to 11.4 ± 0.8% in MLD at 26 dpn and 10.2 ± 1.1% in MST. There was a supplementation effect in MLD (*p* = 0.01) indicating a higher percentage of PAX7+ nuclei in Gln supplemented pigs at 5 dpn independent of birth weight.

**Figure 2:**
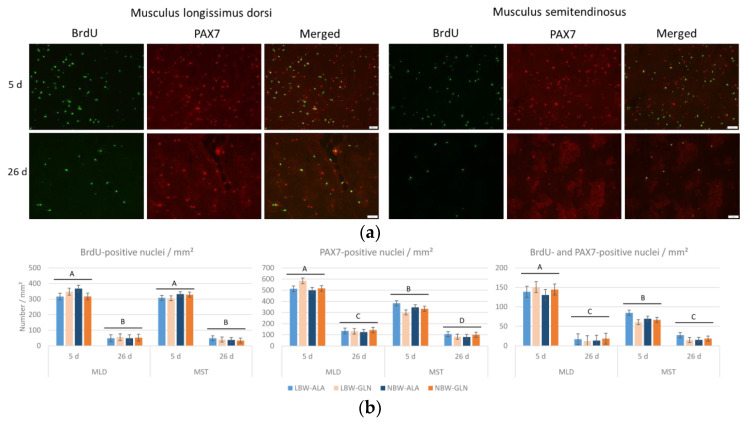
Co-localization of BrdU and PAX7 in muscle cross-sections of piglets at 5 and 26 dpn (*n* = 12 per group). (**a**) Fluorescence images of BrdU (green) and PAX7 (red) and the overlay of both images, where co-localization appears yellow. Scale bar represents 20 µm. (**b**) Quantification of BrdU+, PAX7+ and BrdU+/PAX7+ nuclei per area unit in two muscles of low-birth-weight (LBW) and normal-birth-weight (NBW) piglets supplemented with glutamine (GLN) or alanine (ALA). Values represent LSmeans with standard errors in error bars. ^A–D^ indicate significant differences among ages and muscles (*p* ≤ 0.05, Tukey–Kramer test).

The number of proliferating cells (BrdU+) and satellite cells (PAX7+) per mm² was much lower in pigs at 26 dpn than at 5 dpn in both muscles (Figure 2). Fewer satellite cells per mm² (Figure 2b) were detected in MST compared to MLD at 5 dpn and 26 dpn (*p* < 0.001 and *p* = 0.04, respectively) and fewer proliferating satellite cells (BrdU+/PAX7+) per mm² were detected in MST than in MLD at 5 dpn (*p* < 0.001). The variability within all groups was high; thus, the influence of neither birth weight nor supplementation on the number of BrdU+, PAX7+ or positive for both was observed (*p* ≥ 0.34).

**Figure 3:**
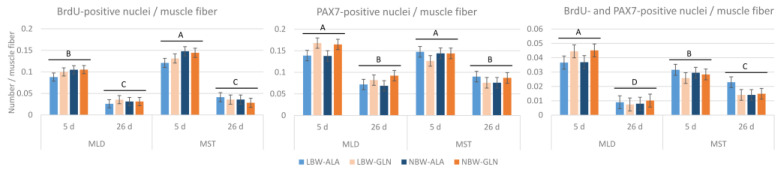
Quantification of BrdU+, PAX7+ and BrdU+/PAX7+ nuclei per muscle fiber in two muscles of low-birth-weight (LBW) and normal-birth-weight (NBW) piglets supplemented with glutamine (GLN) or alanine (ALA). Values represent LSmeans with standard errors in error bars (*n* = 12 per group). ^A–D^ indicate significant differences among ages and muscles (*p* ≤ 0.05, Tukey–Kramer test).

Related to the number of muscle fibers (Figure 3), there were more proliferating cells in MST than in MLD at 5 dpn (*p* < 0.001), but fewer proliferating satellite cells (*p* < 0.001). The numbers of proliferating cells, satellite cells and proliferating satellite cells all decreased with age (*p* < 0.001). Additionally, there was a supplementation × muscle interaction effect (*p* = 0.008) for the number of PAX7+ cells, indicating more satellite cells in MLD with Gln supplementation independent of age. Similarly, there was a supplementation × muscle interaction effect (*p* = 0.009) for the number of BrdU+/PAX7+ cells and, hence, more proliferating satellite cells in MLD of Gln supplemented pigs.

**Figure 4:**
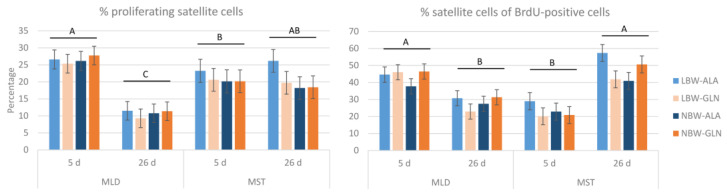
Quantification of the percentage of BrdU+ satellite cells and the percentage of PAX7+ proliferating cells in two muscles of low-birth-weight (LBW) and normal-birth-weight (NBW) piglets supplemented with glutamine (GLN) or alanine (ALA). Values represent LSmeans with standard errors in error bars (*n* = 12 per group). ^A–C^ indicate significant differences among ages and muscles (*p* ≤ 0.05, Tukey–Kramer test).

The percentage of satellite cells undergoing proliferation was reduced from about 26% to 11% in MLD at 26 dpn compared to 5 dpn (*p* < 0.001), whereas it remained nearly unchanged in MST (*p* ≥ 0.99; Figure 4). Birth weight and supplementation had no effect (*p* ≥ 0.41). The percentage of satellite cells from all proliferating cells decreased from about 44% at 5 dpn to about 28% at 26 dpn in MLD (*p* < 0.001), whereas it increased from about 23% to 47% in MST (*p* < 0.001; Figure 4). No significant influence of birth weight or supplementation was detected (*p* ≥ 0.37).

**Figure 5:**
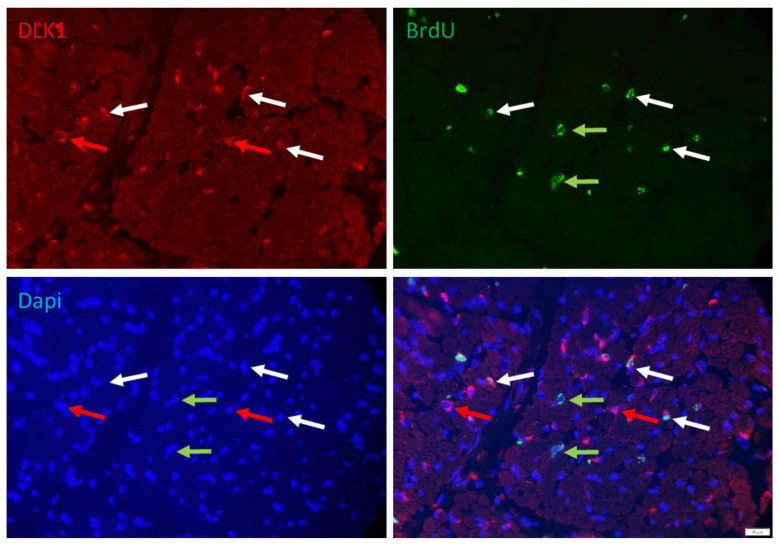
Co-localization of BrdU and DLK1 in a muscle cross-section of a piglet at 5 dpn. Fluorescence images of DLK1 (red), BrdU (green) and Dapi nuclear stain (blue) and the merged image. Arrows indicate examples of cells that are DLK1+ (red), BrdU+ (green) or DLK1+/BrdU+ (white). Scale bar represents 20 µm.

More than 50% of the proliferating cells were apparently non-satellite cells. They could be stem cells not yet committed to the myogenic or adipogenic lineage. Those cells were identified with DLK1 as a marker. The signal of DLK1 could be detected in the cytoplasm of numerous interstitial cells in the muscle cross-section (Figure 5). A portion of them was also BrdU+ and may represent another interesting class of cells, namely, preadipocytes. However, the location of many of those cells between muscle fibers also suggested that cell types other than preadipocytes are DLK1+.

**Figure 6:**
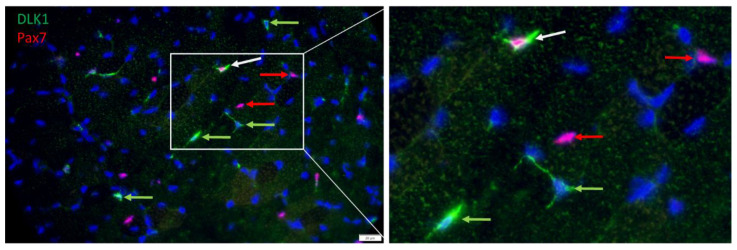
Co-localization of PAX7 (red) and DLK1 (green) in a muscle cross-section of a piglet at 26 dpn. All nuclei were stained with Hoechst 33258 (blue). The three fluorescence channels are merged. The framed part of the left image is shown magnified in the right image. Arrows indicate examples of cells that are DLK1+ (green), PAX7+ (red) or DLK1+/PAX7+ (white). Scale bar represents 20 µm.

Serial immunostaining with antibodies against PAX7 and DLK1, confirmed that satellite cells were among DLK1+ cells. The nuclear localization of PAX7 and cytoplasmic localization of DLK1 (Figure 6) indicated independent signals without cross-reactions of the antibodies. There were cells either positive for PAX7 or for DLK1, but also some PAX7+/DLK1+ cells. Thus, a portion of the PAX7+ cells were also DLK1+ and vice versa. Due to this partial overlap between PAX7+ and DLK1+ cells, it was not possible to independently quantify the number of proliferating preadipocytes.

**Figure 7:**
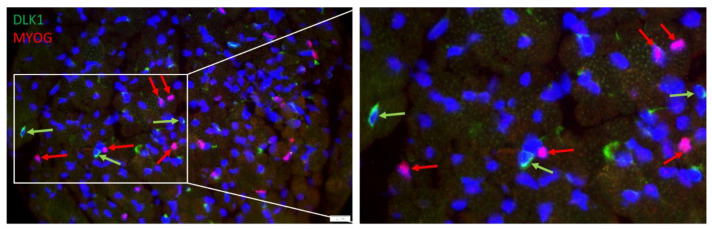
Double staining of Myogenin (red) and DLK1 (green) in a muscle cross-section of a piglet at 5 dpn. All nuclei were stained with Hoechst 33258 (blue). The three fluorescence channels are merged. The framed part of the left image is shown magnified in the right image. Arrows indicate examples of cells that are DLK1+ (green) or PAX7+ (red). Scale bar represents 20 µm.

However, myogenic cells that were more advanced in their development and expressed myogenin were negative for DLK1, as shown by serial immunostaining with respective antibodies. Again, myogenin was localized in the cell nucleus, whereas DLK1 was localized in the cytoplasm of different cells (Figure 7), indicating independent staining patterns. Both proteins were not detected in the same cells in muscle tissue of piglets.

**Figure 8:**
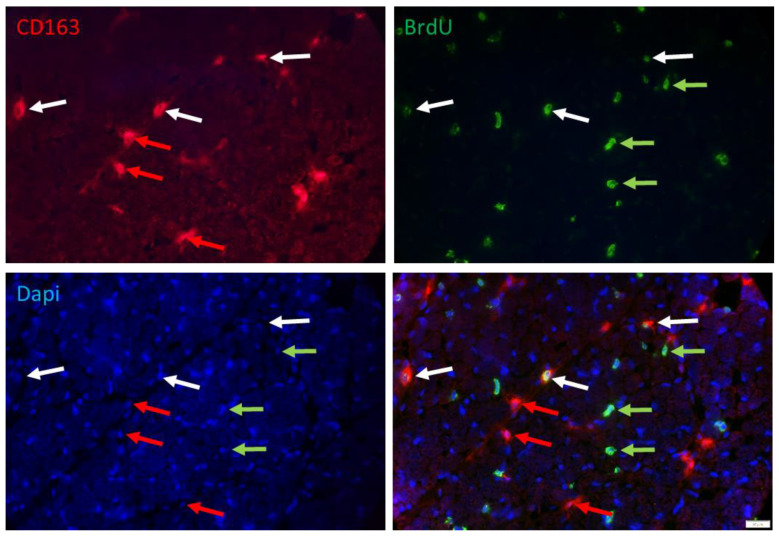
Double staining of BrdU and CD163 in a muscle cross-section of a piglet at 5 dpn. Fluorescence images of CD163 (red), BrdU (green) and Dapi nuclear stain (blue) and the merged image. Arrows indicate examples of cells that are CD163+ (red), BrdU+ (green) or CD163+/BrdU+ (white). Scale bar represents 20 µm.

Another class of cells that are proliferating in muscle tissue of young pigs could be immune cells. Double immunostaining with BrdU and macrophage marker CD163 (Figure 8) and quantification in a subsample (*n* = 3 per group) confirmed that 34.3% and 39.9% of the BrdU+ cells were immune cells at 5 and 26 dpn, respectively. There was no effect of birth weight or supplementation (*p* > 0.3) on the percentage of macrophages among the BrdU+ cells. However, the percentage of proliferating cells among macrophages decreased from 40.7% at 5 dpn to 18.8% at 26 dpn (*p* < 0.001), without any effect of birth weight or supplementation.

**Figure 9:**
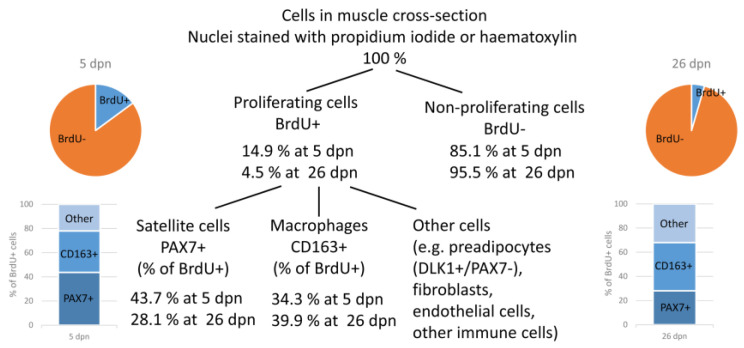
Overview of quantified and/or identified proliferating cells in muscle cross-sections of piglets at 5 and 26 dpn. Values are means of the respective ages, independent of birth weight or supplementation.

An overview of the quantification of proliferating cells in muscle tissue of piglets at 5 or 26 dpn, independent of birth weight or supplementation, is shown in Figure 9. Of note, the category “other” comprises different cell types that could not be quantified separately. They were calculated as difference between the percentage of quantified satellite cells and macrophages and 100%.

## 4. Discussion

Many studies on glutamine supplementation in piglets focused on effects on the intestinal tract and more general effects on growth and health of the animals [29,30,31]. Although some studies reported positive effects on piglet growth when supplemented with glutamine [32,33], a direct effect of supplementation on skeletal muscle was barely investigated. The growth promotion by Gln supplementation in piglets during the suckling phase was explained by improved intestinal health [34], and thus, a better overall nutrient availability could be suggested. However, the piglets in our study did not differ in jejunal development during the suckling phase, as reported by Schregel et al. [27]. Nevertheless, we have recently shown that oral glutamine supplementation starting from birth had some minor effects on skeletal muscle morphology and on gene expression, especially in LBW piglets [4]. A subsequent study suggested increased proliferation in muscle tissue of Gln supplemented LBW piglets during the early postnatal phase and indicated that Gln can stimulate proliferating satellite cells from those animals in muscle cell culture [8]. This could have a long-term effect by improving muscle growth.

Here, we aimed at quantification of proliferating muscle satellite cells in response to Gln supplementation and at the identification of further affected cell types in skeletal muscle tissue of larger groups of suckling piglets. We provide the first study of sufficient size (*n* = 12 per group) allowing for statistically valid conclusions. Comparing the investigated muscles, we recorded a higher percentage of proliferating cells in MST than in MLD, which may indicate faster development of leg muscles during the early postnatal phase in accordance with observed larger muscle fibers [4]. However, the number of proliferating cells was not influenced by supplementation or birth weight. Different cell types proliferate in postnatal muscle tissue that could be affected differently by supplementation. Since satellite cells contribute directly to muscle fiber growth, they were quantified in this study using PAX7 as a well-established marker for satellite cells [35]. As expected, nuclei of skeletal muscle satellite cells comprise a large fraction of total nuclei (20–25%) in skeletal muscle of early postnatal piglets. Their number decreased by 50 to 70% within three weeks, indicative of a rapid development of the muscles. At day 5, we observed a positive effect of Gln supplementation on the number of satellite cells independent from birth weight in MLD but not MST. This suggests a compensatory effect of the supplementation on retarded maturation of this muscle in the first days after birth. However, the differential effect on muscle cells cultured from low- and normal-birth-weight piglets [8] was not observed in vivo.

Among the total number of proliferating cells, muscle satellite cells comprise a proportion of about 50%, making it to the largest fraction. On the other hand, this implicates that a large number of non-myogenic cells are proliferating in the skeletal muscle of piglets during the suckling period. The decline in the number of proliferating satellite cells with age was proportional to the decrease in the number of total proliferating cells. The number of remaining satellite cells per mm² was significantly higher in MLD than in MST muscle at 26 dpn, due to the larger muscle fibers, but a similar number of satellite cells were in a proliferative state. Thus, we observed more proliferating satellite cells per muscle fiber in MST at 26 dpn. Together with the higher percentage of satellite cells among proliferating cells, this suggests persistent muscle development and indicates a higher growth potential of the MST at this later age. This assumption is supported by results from Berard et al. [36] showing that satellite cells contribute to the elongation of existing as well as to the formation of new muscle fibers in piglets during the suckling period. We found a positive effect of Gln supplementation on the fraction of proliferating satellite cells only in MLD of young piglets, but an increase in apparent muscle fiber number from 5 to 26 dpn in both muscles [4]. Thus, the results support, on the one hand, a persisting muscle fiber formation and elongation process and, on the other hand, that muscles were differently affected by Gln supplementation and develop differently.

The results indicated that more than 50% of the proliferating cells were apparently non-satellite cells. Among them could be stem cells with the ability to differentiate in the myogenic or adipogenic direction, but also endothelial cells, fibroblasts, immune cells and others. While an earlier study demonstrated that DLK1 could be a preadipocyte marker in cattle muscle tissue [18], the staining pattern in pig muscle tissue showed many positive cells between muscle fibers and few in connective tissue. Thus, in contrast to satellite cell specific PAX7, DLK1 marks a more heterogeneous cell population. We observed cells that were positive for BrdU and DLK1 most likely marking proliferating preadipocytes located in the perimysium of skeletal muscle tissue. However, there were several cells positive for DLK1 and PAX7 located in the endomysium. This points to the dual role of DLK1 as adipogenesis inhibitor and regulator of the myogenic program in muscle development [23]. As shown by Fu et al. [24], porcine DLK1 is highly expressed during pig embryogenesis and can regulate satellite cell proliferation, differentiation and fusion with muscle fibers. The expression of DLK1 declines gradually during the first weeks after birth in pigs [37]. Thus, the DLK1+/PAX7+ cells in muscle tissue of piglets might comprise early stages of satellite cells, and DLK1+/PAX7− cells may be stem cells not yet finally committed to either adipogenic or myogenic differentiation. This was indirectly confirmed by staining with myogenin—a marker for advanced differentiation of muscle cells, which was found exclusively in cells negative for DLK1. Olguin and Olwin [38] have shown in cell cultures that PAX7 and myogenin expression is mutually exclusive; thus, myogenin was only detected when PAX7 was down-regulated. This may apply for DLK1 and myogenin as well, but this has to be verified.

Immune cells are involved in muscle repair and regeneration after injury as reviewed by Ziemkiewicz et al. [39]. Infiltrating immune cells usually have a short retention time, but subtypes of macrophages reside in skeletal muscle tissue and interact with muscle stem cells [39]. Arnold et al. [40] demonstrated in co-cultures that pro-inflammatory M1 macrophages enhanced myogenic precursor cells proliferation, whereas anti-inflammatory M2 macrophages enhanced differentiation. On the other hand, macrophages are known to inhibit proliferation of adipogenic progenitors [41] and may play an important role in regulation of muscle tissue composition. As one example for immune cells in the growing muscle, we identified resident macrophages and could confirm that the CD163-positive M2 macrophages comprise a substantial fraction of proliferating cells in early postnatal muscle tissue. Their role should be further elucidated.

In summary, we have experimentally demonstrated that satellite cells comprise the largest fraction of cells proliferating in skeletal muscle of piglets early after birth. The second largest fraction are macrophages at this time point. In contrast, this fraction was largest at weaning, while the absolute numbers decreased. An early, oral Gln supplementation of piglets has limited positive effects on skeletal muscle development at cellular level, in line with our previous results [4,8]. The main effect is satellite cell activation. However, this effect was independent of the birth weight of the piglets. Thus, Gln supplements may not be considered as effective treatment in piglets with low birth weight regarding growth, but they may be further used for promoting piglets’ intestinal integrity in stressful situations [33] and, with that, supporting a better overall nutrient supply.

## 5. Conclusions

An early, oral Gln supplementation of piglets had limited positive effects on skeletal muscle development, in particular, on satellite cell activation. Since this effect was independent of the birth weight of the piglets, Gln supplementation may not be considered an effective treatment to promote the growth of piglets with low birth weight.

## Data Availability

Data presented in this manuscript are available upon reasonable request to the corresponding author.

## Supplementary Materials

The following supporting information can be downloaded at: https://www.mdpi.com/2073-4409/12/4/580/s1, Figure S1: Negative control for double immunostaining with antibodies against PAX7 and BrdU in a muscle cross-section of a piglet at 5 dpn; Figure S2: Immunohistochemical detection of BrdU (green) and PAX7 (red) in a muscle cross-section of *Musculus longissimus dorsi*; Figure S3: Negative control for double immunostaining with antibodies against CD163 and BrdU in a muscle cross-section of a piglet at 5 dpn; Figure S4: Negative controls for detection of unspecific binding of secondary antibodies in porcine muscle cross-sections.
