# Supplementary material for: Identification and Quantification of Proliferating Cells in Skeletal Muscle of Glutamine Supplemented Low- and Normal-Birth-Weight Piglets
Source: Cells. 2023 Feb 11;12(4):580. doi: 10.3390/cells12040580 (PMC9953894; doi:10.3390/cells12040580)
Supplement: Supplementary file 1 [file cells-12-00580-s001.zip › cells-2141287-supplementary.pdf]

# Supplementary Material

Supplementary Figure S1:

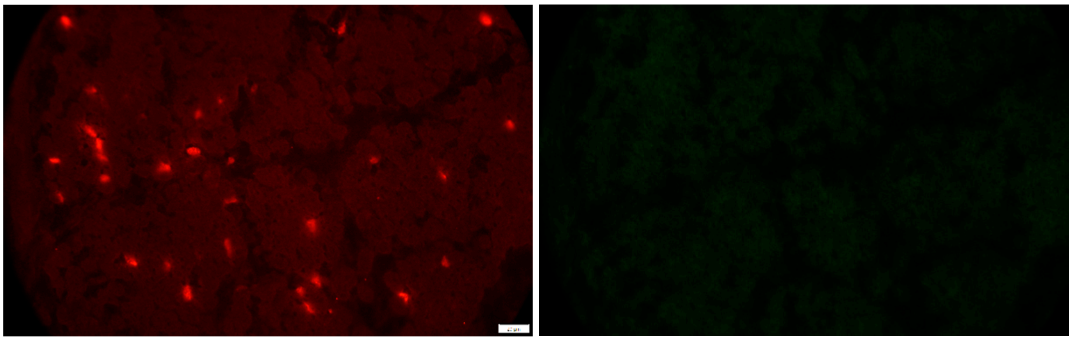

**Figure S1.** Negative control for double immunostaining with antibodies against PAX7 and BrdU in a muscle cross-section of a piglet at 5 dpn. Left: Immunohistochemical detection of PAX7 with a mouse anti-PAX7 antibody (DSHB, Univ. Iowa, USA) and an Alexa Fluor 594 goat anti-mouse IgG (H+L) secondary antibody (red). Right: The subsequent incubation with Alexa Fluor 488 goat anti-mouse IgG1 (green), which was used for BrdU detection, did not generate an identical fluorescence signal. This indicates no disturbing interference between signals for PAX7 and BrdU. Scale bar represents 20 µm.

Supplementary Figure S2:

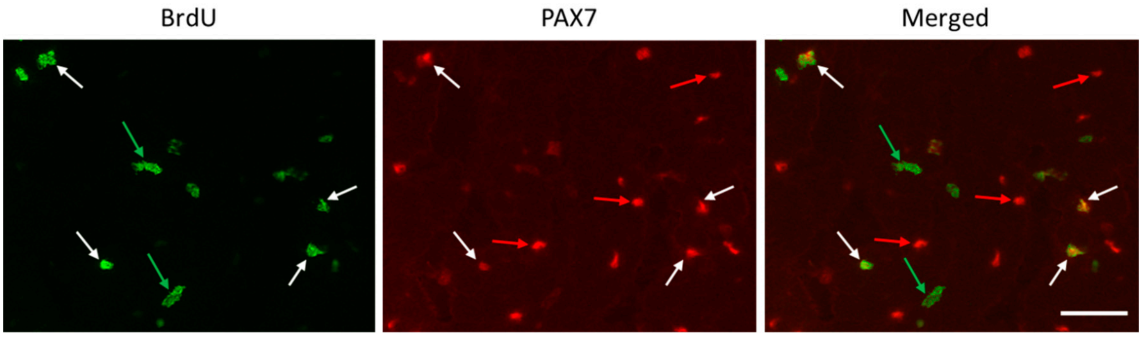


**Figure S2.** Immunohistochemical detection of BrdU (green) and PAX7 (red) in a muscle cross-section of *Musculus longissimus dorsi*. Arrows indicate examples for nuclei that were either BrdU+ (green arrows) or PAX7+ (red arrows) or positive for both (white arrows), indicating specificity and no cross-reactivity of the antibodies, as a prerequisite for the subsequent analysis. Scale bar represents 20 µm.

Supplementary Figure S3:

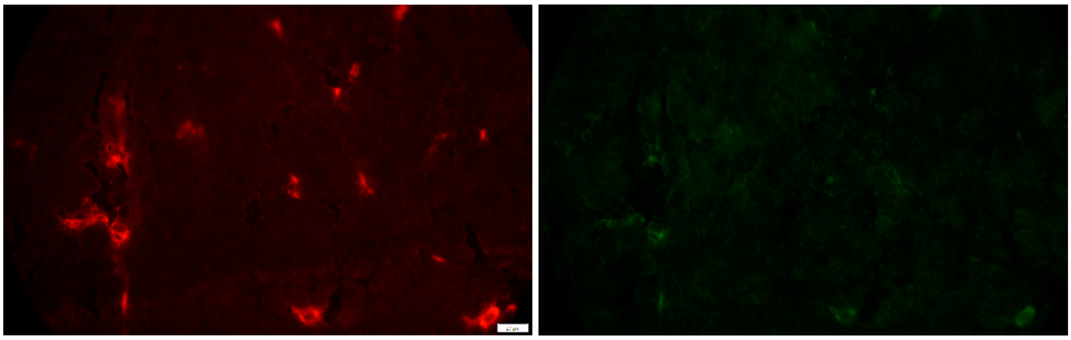

**Figure S3.** Negative control for double immunostaining with antibodies against CD163 and BrdU in a muscle cross-section of a piglet at 5 dpn. Left: Immunohistochemical detection of CD163 with a mouse anti-CD163 antibody (AbD Serotec, BioRad, Munich, Germany) and an Alexa Fluor 594 goat anti-mouse IgG (H+L) secondary antibody (red). Right: The subsequent incubation with Alexa Fluor 488 goat anti-mouse IgG1 (green),

which was used for BrdU detection, generated only a very weak fluorescence signal. This indicates no disturbing interference between signals for CD163 and BrdU.

Supplementary Figure S4:

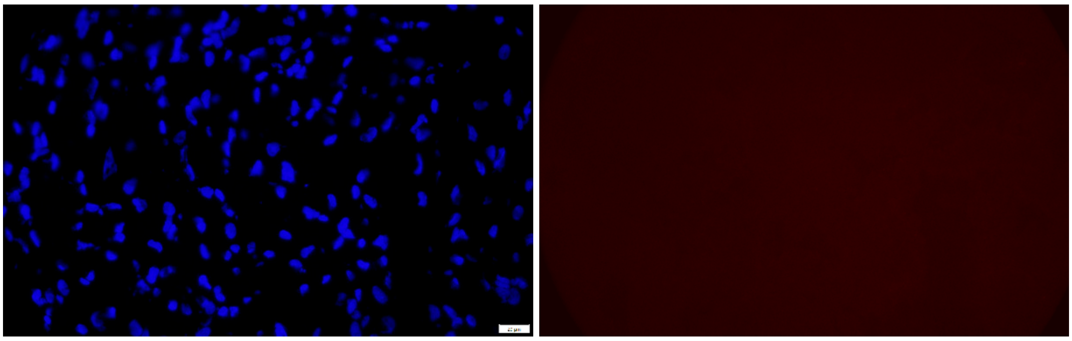

(a)

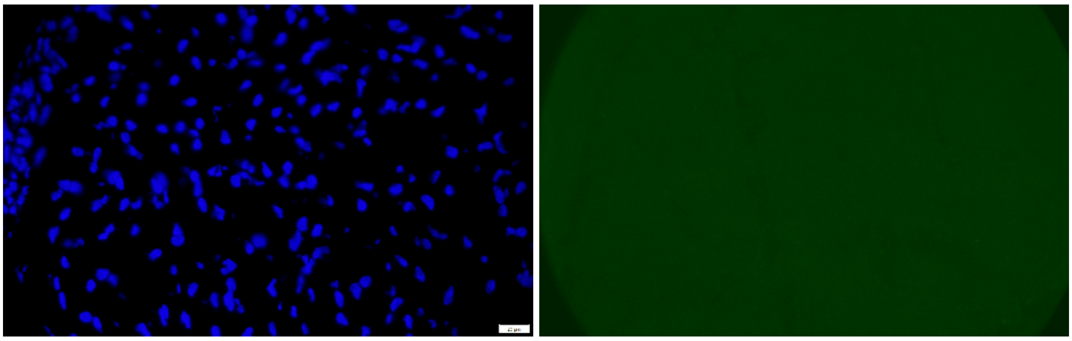

(b)

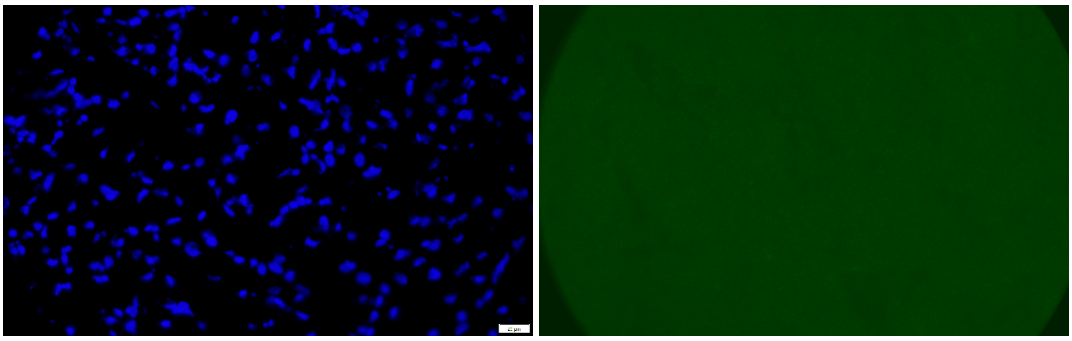

(c)

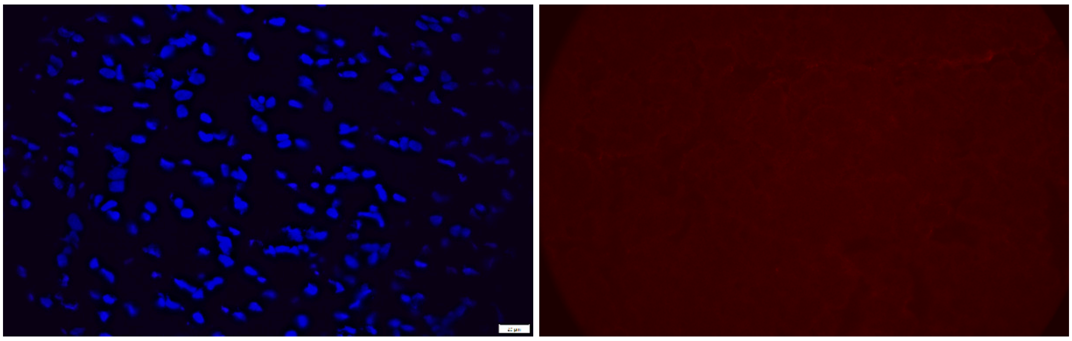

(d)

**Figure S4.** Negative controls for detection of unspecific binding of secondary antibodies in porcine muscle cross-sections. Primary antibodies were replaced with normal goat serum. (a) Alexa Fluor 594 goat anti-mouse IgG (H+L); (b) Alexa Fluor 488 goat anti-mouse IgG1; (c) Alexa Fluor 488 goat anti-rabbit IgG; (d) Alexa Fluor 594 goat anti-rabbit IgG . Nuclei were counterstained with Dapi. Scale bars represent 20 µm.
